# Solar energy prediction through machine learning models: A comparative analysis of regressor algorithms

**DOI:** 10.1371/journal.pone.0315955

**Published:** 2025-01-02

**Authors:** Huu Nam Nguyen, Quoc Thanh Tran, Canh Tung Ngo, Duc Dam Nguyen, Van Quan Tran

**Affiliations:** 1 Institute for Hydropower and Renewable Energy, Vietnam Academy for Water Resources, Hanoi, Vietnam; 2 Hydraulic Construction Institute, Vietnam Academy for Water Resources, Hanoi, Vietnam; 3 University of Transport Technology, Hanoi, Vietnam; 4 Institute of Training and International Cooperation (ITIC), University of Transport Technology, Hanoi, Vietnam; UCSI University Kuala Lumpur Campus: UCSI University, MALAYSIA

## Abstract

Solar energy generated from photovoltaic panel is an important energy source that brings many benefits to people and the environment. This is a growing trend globally and plays an increasingly important role in the future of the energy industry. However, it intermittent nature and potential for distributed system use require accurate forecasting to balance supply and demand, optimize energy storage, and manage grid stability. In this study, 5 machine learning models were used including: Gradient Boosting Regressor (GB), XGB Regressor (XGBoost), K-neighbors Regressor (KNN), LGBM Regressor (LightGBM), and CatBoost Regressor (CatBoost). Leveraging a dataset of 21045 samples, factors like Humidity, Ambient temperature, Wind speed, Visibility, Cloud ceiling and Pressure serve as inputs for constructing these machine learning models in forecasting solar energy. Model accuracy is meticulously assessed and juxtaposed using metrics such as coefficient of determination (R^2^), Root Mean Square Error (RMSE), and Mean Absolute Error (MAE). The results show that the CatBoost model emerges as the frontrunner in predicting solar energy, with training values of R^2^ value of 0.608, RMSE of 4.478 W and MAE of 3.367 W and the testing value is R^2^ of 0.46, RMSE of 4.748 W and MAE of 3.583 W. SHAP analysis reveal that ambient temperature and humidity have the greatest influences on the value solar energy generated from photovoltaic panel.

## 1. Introduction

The growing global energy demand, along with the need for clean and sustainable energy sources, has led to a significant increase in solar energy projects worldwide. However, one of the major challenges facing the solar industry is the unpredictability of solar energy production, which is highly dependent on weather conditions such as cloud cover, rainfall, and intensity sunlight. Therefore, developing accurate and reliable models to forecast the power output of solar energy projects is essential, which is important for the effective management of energy systems. In fact, solar energy is expected to play a key role in the global transition to clean and renewable energy sources. The International Energy Agency (IEA) estimates that solar energy could provide up to 30% of the world’s electricity by 2050 [1]. This forecast highlights the need for robust solar forecasting models that can support effective integration of solar energy into the grid and optimization of energy systems. Furthermore, the need for solar energy forecasting is especially urgent in developing countries like Vietnam, where solar energy projects are on the rise and energy demand is growing rapidly. According to Vietnam’s National Power Development Plan, the country’s electricity demand is expected to rise significantly with strong economic growth, reaching around 124,000 MW by 2030 [2]. However, the country is currently heavily dependent on fossil fuels, which not only contributes to greenhouse gas emissions but also exposes the country to fluctuations in global oil prices. Therefore, there is an increasing need to diversify the country’s energy structure, with solar energy being a promising alternative.

Vietnam Electricity Group (EVN) said that in April 2022, the entire system’s electricity production reached 22.62 billion kWh, an increase of 1.9% over the same period. Cumulatively in the first 4 months of the year, the total electricity output of the entire system reached 85.65 billion kWh, an increase of 6.2% over the same period in 2021. Notably, renewable energy including wind power, solar energy, and biomass power reached 13.15 billion kWh, accounting for 15.4% of the total electricity produced in the entire system.

**Fig 1:**
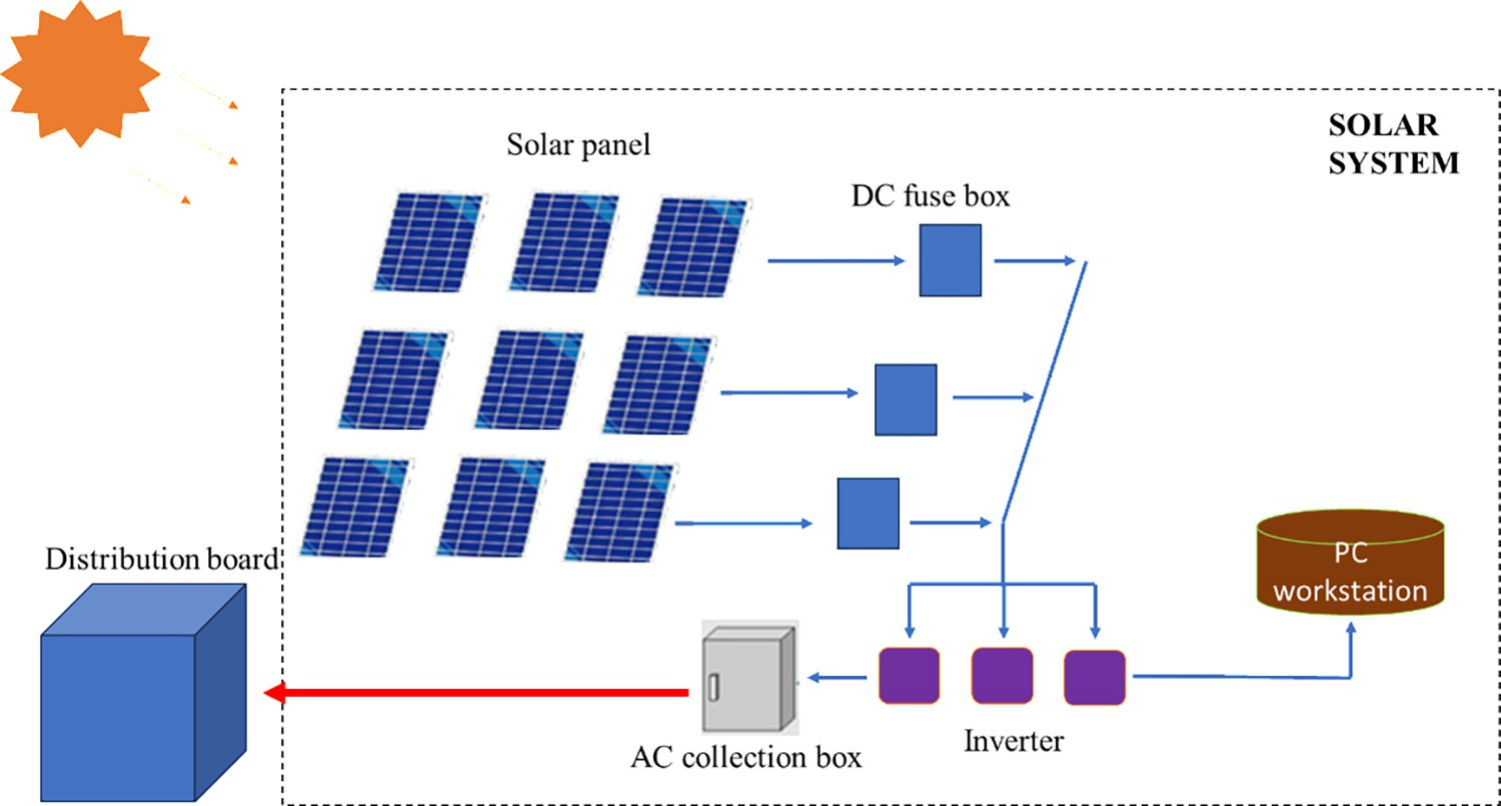
Photovoltaic technology (SPV—Solar photovoltaic).

In recent years, Vietnam has made significant strides in promoting solar energy, with the government implementing policies to encourage the development of solar energy projects. In 2019, Vietnam started construction of the largest solar energy plant in Southeast Asia, with a capacity of 688 MW. The plant is expected to produce about 1.2 billion kWh of electricity annually, enough to power 1.3 million households and reduce 1.2 million tons of carbon emissions each year. The success of this project highlights the potential of solar energy in Vietnam and the need for accurate forecasting models to support effective management of energy systems. According to Draft Power Plan VIII, it is expected that the installed capacity of solar energy will increase from 17 GW (2020–2025 period) to about 20 GW (2030). The proportion of solar energy is expected to account for 17% (2025), 14% (2030) in the structure of power sources. In Vietnam, technology, techniques and the ability to develop solar energy projects are still heavily dependent on foreign countries, leading to large-scale solar energy deployment facing many difficulties, especially about price. This makes it difficult for solar energy to compete with other traditional power sources. The most important application of solar energy today and in the future is still electricity production. Two types of solar energy production technology are widely developed: photovoltaic technology (SPV—Solar photovoltaic) and concentrated solar energy technology (CSP—Concentrated solar energy). The most popular SPV technology today includes: crystalline solar cells (about 90% market share), the rest are thin film solar cells (about 10% market share). Currently in Vietnam, solar energy development investment projects use mostly Solar photovoltaic technology as described in Fig 1. However, evaluating and designing solar cell energy using this technology Solar photovoltaic in Vietnam still has many limitations, mainly due to foreign consulting units. It would be very meaningful if we could make a preliminary assessment of the solar cell energy source.

Implementing solar energy as a significant energy resource presents challenges due to the inherent uncertainty in electricity production, which is highly dependent on weather conditions. To maximize efficiency, it is essential to connect solar plants to the central electricity transmission grid. Accurate forecasting of solar energy production at specific plants is crucial to managing this uncertainty and ensuring smooth electricity transmission [3]. Extensive research has been conducted on solar photovoltaic power forecasting.

Machine learning (ML) has emerged as a powerful tool across various scientific disciplines, enabling accurate predictions, efficient optimization, and deeper insights into complex phenomena. In material science, Jain et al. [4] conducted a comparative analysis of ML techniques to predict the wear and friction properties of MWCNT-reinforced PMMA nanocomposites, demonstrating the effectiveness of these models in material property evaluation. Similarly, Jain et al. [5] applied ML to optimize terahertz metamaterial absorbers, showcasing its potential in enhancing design efficiency. The versatility of ML extends to electrical characterization, as illustrated by Vaja et al. [6], who used it to evaluate the electrical properties of methylene blue solutions via AC/DC conductivity. Furthermore, Prakash et al. [7] reviewed ML’s transformative role in advancing functional materials, particularly single-crystal perovskite halides, from crystal growth to device applications.

Beyond material science, ML’s utility is evident in other domains. In hydrology, Hayder et al. [8] employed NARX neural networks and LSTM-based deep learning to achieve multi-step-ahead river flow predictions, emphasizing its ability to model dynamic natural systems. In geotechnical engineering, Solihin et al. [9] utilized stacking ensemble ML techniques for landslide susceptibility mapping, underscoring its significance in environmental risk management. Additionally, Solihin et al. [10] applied stacked ensemble ML models to calibrate spectroscopy data, revealing its importance in refining analytical techniques.

Together, these studies highlight the extensive applicability of ML in addressing challenges across diverse fields, including material science, electronics, environmental engineering, and analytical spectroscopy, paving the way for more efficient and innovative solutions.

These ML models utilize sophisticated algorithms to analyze various factors including weather conditions, solar panel efficiency, and geographical location [11]. By harnessing historical data alongside real-time weather information, machine learning models can deliver precise and dependable predictions of solar energy output. This capability empowers energy managers to optimize energy systems effectively, leading to reduced operating costs and enhanced efficiency. The use of ML in solar forecasting has attracted significant attention in recent years, with several studies demonstrating the potential of ML-based models to improve accuracy and reliability of solar forecasts.

Adaptive agent decision models based on deep reinforcement learning and autonomous learning have been developed to address complex decision problems such as solar energy forecasting [12]. For example, these models have been applied to neurophysiological data, demonstrating their applicability to real-world solar energy forecasting challenges [13].

Lorenz et al. [14] provided a comprehensive overview of the field, while Raza et al. [15] highlighted recent advancements. Many studies in this area focus on predicting irradiance or leveraging historical power output data. For instance, Yang et al. [16] used exponential smoothing to improve predictions of horizontal irradiance, and Gueymard [17] examined irradiance forecasting for surfaces at various angles. Lorenz et al. [18] utilized regional weather data to predict irradiance, which was then converted into power forecasts. Several studies have explored the use of weather data and historical power output to predict both irradiance and power output [19, 20].

Daily mean solar irradiance is a critical factor in determining the size of solar energy generation units. Accurate forecasting of solar irradiation at specific locations aids in predicting the electricity output of solar panels, which is vital for calculating system size, return on investment (ROI), and load measurements. Various regression algorithms have been applied in conjunction with solar irradiance parameters to improve the accuracy of these forecasts [21].

Gonzalez et al. [22] proposed an ML-based forecasting model that uses machine learning algorithms to predict the hourly solar energy output of photovoltaic systems electricity. The ML model achieved 94.9% accuracy in predicting solar energy output, outperforming traditional forecasting methods [23]. Ortiz et al. [24] proposed an ML-based model using deep learning algorithms to forecast the power output of solar energy plants. The ML model leverages real-time weather data, historical solar output data, and plant operating data to provide accurate and reliable forecasts of solar output. The ML model achieved over 90% accuracy in predicting solar energy output, demonstrating the potential of ML-based models in improving the efficiency and reliability of energy systems.

The variability of solar radiation often leads to a mismatch between energy demand and supply, highlighting the need for efficient thermal energy storage systems. These systems are crucial for bridging the gap and enabling solar thermal power plants to provide uninterrupted power generation to meet both current and future energy needs. Anand et al. [25] employed popular machine learning models including K-nearest neighbors (KNN) and extreme gradient boosting (XGBoost)—to evaluate the performance of a packed-bed thermal energy storage system. Aksoy and Genc [26] used three boosting models including XGBoost, Light Gradient Boosting (LightGBM) and CatBoost for forecasting the power energy to be generated by solar energy plants. Krishnan et al. [27] used Gradient boosting (GB) for forecasting solar ration in various climatic zones. However, the use of machine learning models or artificial intelligence (AI) models in solar energy forecasting is still in its infancy, with limited research and practical applications in Vietnam.

Therefore, five machine learning models including XGBoost, LightGBM, GB, CatBoost and KNN will be introduced for building five ML models in predicting power solar cell capacity from six input variables including Humidity, Ambient temperature, Wind Speed, Visibility, Pressure, and Cloud Ceiling.

## 2. Significance of the investigation

The primary objective of this study is to develop and evaluate the performance of five machine learning models XGBoost, LightGBM, Gradient Boosting (GB), CatBoost, and KNN for predicting solar energy output. By incorporating six key weather-related input variables (humidity, ambient temperature, wind speed, visibility, pressure, and cloud ceiling), the study aims to improve forecasting accuracy, facilitating effective energy management and integration of solar power into the electricity grid.

This study contributes to the growing body of research on solar energy forecasting by:—Demonstrating the application and comparative performance of five machine learning models in predicting solar power generation, with CatBoost emerging as the best-performing model.

- Employing SHAP analysis and partial dependence plots to uncover the relative importance and non-linear interactions of input variables, such as ambient temperature and humidity, on solar energy output.
- Identifying critical gaps in the dataset, including the absence of photovoltaic panel-specific technical data, and discussing their impact on model accuracy.
- Emphasizing the need for larger, more diverse datasets and the inclusion of solar technology specifications to enhance prediction reliability and model generalizability. These contributions advance the understanding of machine learning applications in renewable energy forecasting and provide a foundation for improving solar energy system efficiency.

## 3. Description of database

In this study, the dataset used to build a machine learning model consists of 21.045 samples of solar energy derived from the investigation of Williams and Wagner [28]. The dataset includes six input variables and one output variable. The input variables include factors such as Humidity, Ambient temperature, Wind Speed, Visibility, Pressure, and Cloud Ceiling depending on the specific context of the study. The output variable could be the amount of energy generated by the solar panels in each sample or a type of energy efficiency index. Statistical values for these variables include: Mean, minimum value, maximum value, std, mean, Median… Detailed information regarding these statistical values can be found in the Table 1 referenced in the study.

**Table 1:** The statistical values of the variables.

No1	Parameter		Count	Mean	Std	Min	Q_25%_	Median	Q_75%_	Max
1	Humidity	%	21045	37.122	23.823	0	17.529	33.124	52.594	99.988
2	Ambient temperature	°C	21045	29.285	12.367	-19.982	21.915	30.289	37.475	65.738
3	Wind Speed	m/s	21045	10.318	6.385	0	6	9	14	49
4	Visibility	m	21045	9.7	1.352	0	10	10	10	10
5	Pressure	kWh	21045	925.945	85.216	781.7	845.5	961.1	1008.9	1029.5
6	Cloud Ceiling	m	21045	515.967	301.903	0	140	722	722	722
7	Power output	W	21045	12.979	7.123	0.257	6.405	13.799	18.864	34.285

This variable serves as the target of prediction or analysis within the machine learning model. Specifically in the realm of solar energy, it typically denotes the quantity of energy produced by solar panels over a certain period. This Power output is commonly quantified in units of Watts (W) or kilowatt-hours (kWh), providing insights into the effectiveness and efficiency of solar energy generation systems. Understanding and accurately predicting this energy output is vital for various applications, including optimizing solar panel placement, assessing system performance, and facilitating energy management strategies [11].

The data used in this study pertains to the utilization of a specific set of input variables, which include: Humidity, temperature, wind speed, visibility, cloud ceiling, pressure.

• Humidity: alters the path of incoming sunlight through phenomena such as refraction, diffraction, and reflection. These optical processes can scatter and disperse sunlight, potentially reducing the intensity of solar radiation reaching the solar panels. Consequently, variations in humidity levels can directly influence the amount of solar energy available for conversion by photovoltaic cells [29].

Indirect Impact on Panel Efficiency: Moreover, humidity indirectly affects the efficiency of solar panels by contributing to the formation of dew. When water vapor in the air condenses on the surface of solar panels as dew, it can enhance the coagulation of dust particles. This increased dust accumulation on the panels can diminish their effectiveness by obstructing sunlight absorption and reducing overall energy output. Therefore, humidity indirectly influences solar panel maintenance requirements and long-term performance [30]. Understanding the interplay between humidity and solar energy generation is essential for optimizing the design, operation, and maintenance of solar energy systems. Incorporating this knowledge into predictive models can help improve the accuracy of energy production forecasts and inform strategic decisions for maximizing solar energy utilization.

- Ambient temperature indeed influences the electrical performance of solar panels. As temperature increases, the efficiency of solar panels tends to decrease, impacting their electrical output. This phenomenon occurs due to several reasons: Decreased Voltage Output: Elevated temperatures can lead to a reduction in the voltage output of solar panels. This decrease happens because the increase in temperature raises the intrinsic carrier concentration of semiconductor materials within the solar cells. Consequently, the built-in voltage of the solar cell diminishes, resulting in a reduction in available voltage output [31, 32].

Elevated Ambient Temperature can induce thermal stress within the materials of solar panels, potentially leading to material degradation and reduced performance over time. Thermal expansion and contraction cycles can cause mechanical stress on the solar cells and interconnects, compromising their structural integrity and electrical performance [33, 34].Understanding the impact of temperature on the electrical performance of solar panels is essential for optimizing the design, operation, and maintenance of solar energy systems. Strategies such as proper panel orientation, ventilation, and thermal management techniques can help mitigate the adverse effects of temperature and maximize the overall energy yield of solar installations.

- Wind speed: Wind speed refers to the rate of movement of air, typically measured in units such as meters per second (m/s) or miles per hour (mph). In the context of solar energy, wind speed significantly impacts the performance of solar panels [35]. wind speed can also affect the sunlight reaching the solar panels. While wind can aid in cleaning their surface, excessively strong winds may create turbulence and cause fluctuations in sunlight. This turbulence can reduce the stability of sunlight hitting the panels, affecting overall system performance [36].
- Visibility can refer to the ability to observe sunlight. High visibility may indicate clear and intense sunlight, while low visibility could suggest that sunlight is obscured or scattered, impacting the efficiency of solar energy generation. In practical terms, high visibility conditions typically mean that there are minimal obstructions, such as clouds, fog, or haze, allowing sunlight to penetrate the atmosphere and reach solar panels with little interference. This results in optimal conditions for solar energy production, as the panels receive a consistent and strong influx of sunlight [36].

Therefore, visibility plays a significant role in determining the performance and output of solar energy systems, with high visibility generally correlating with improved energy production and low visibility indicating potential challenges for solar energy generation.

- Pressure refers to the force exerted on a unit area, typically measured in units such as Pascals (Pa) or atmospheres (atm). It plays a significant role in various contexts, such as influencing weather systems that affect wind patterns or impacting the structural integrity in engineering. Pressure, while not as directly influential as factors like sunlight or temperature, can still impact solar energy production in several indirect ways. Changes in atmospheric pressure can alter the density of the air. Denser air can scatter and absorb more sunlight before it reaches the solar panels, potentially reducing the amount of solar radiation that actually hits the panels.

In some cases, atmospheric pressure might influence the cooling efficiency of solar panels. Lower atmospheric pressure at higher altitudes can lead to less effective convective cooling, which might increase the operating temperature of the panels and reduce their efficiency.

- Cloud Ceiling is a term commonly used in aviation and meteorology to describe the height from the ground to the base of the lowest layer of clouds. It is typically measured in meters or feet. When this height is low, it indicates that clouds are forming close to the ground or that there are thick and low clouds. Conversely, when this height is high, it means that clouds are at a higher altitude and do not pose significant obstruction to observation or aviation activities [37, 38].

In the context of solar energy, cloud ceiling can also refer to the height of clouds measured at a weather station or in a specific area. Information about cloud ceiling can be used to predict the intensity of sunlight and the impact of clouds on solar energy generation in a particular area.

**Fig 2:**
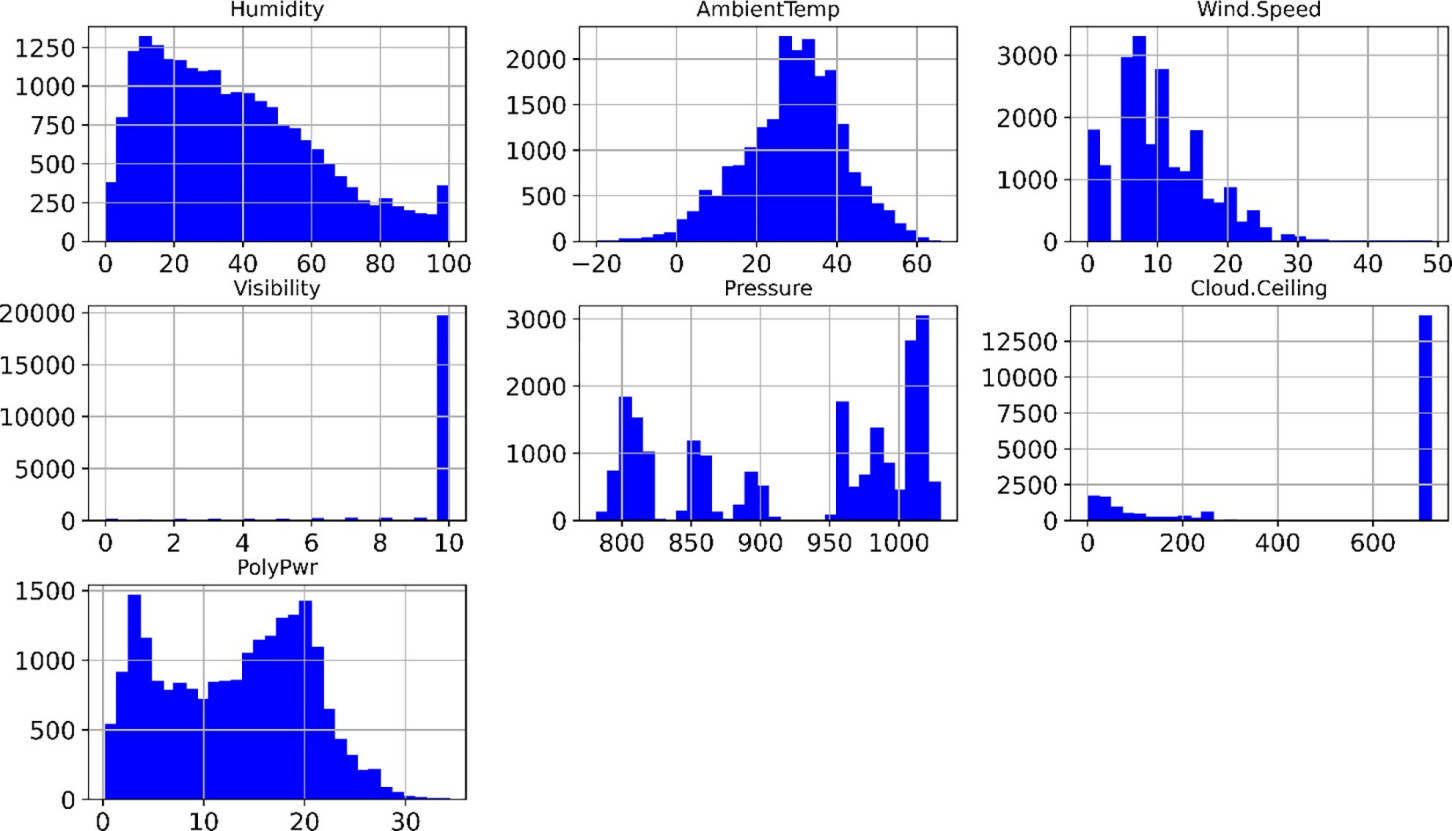
Statistical analysis distribution of the inputs in this study.

To describe the detailed distribution of the input variables with the output variable using histograms Fig 2. By plotting histograms of the input variables and the output variable, we can compare their distributions and understand the relationship between them. This provides us with an overall view of the data and prepares us for building machine learning models.

**Fig 3:**
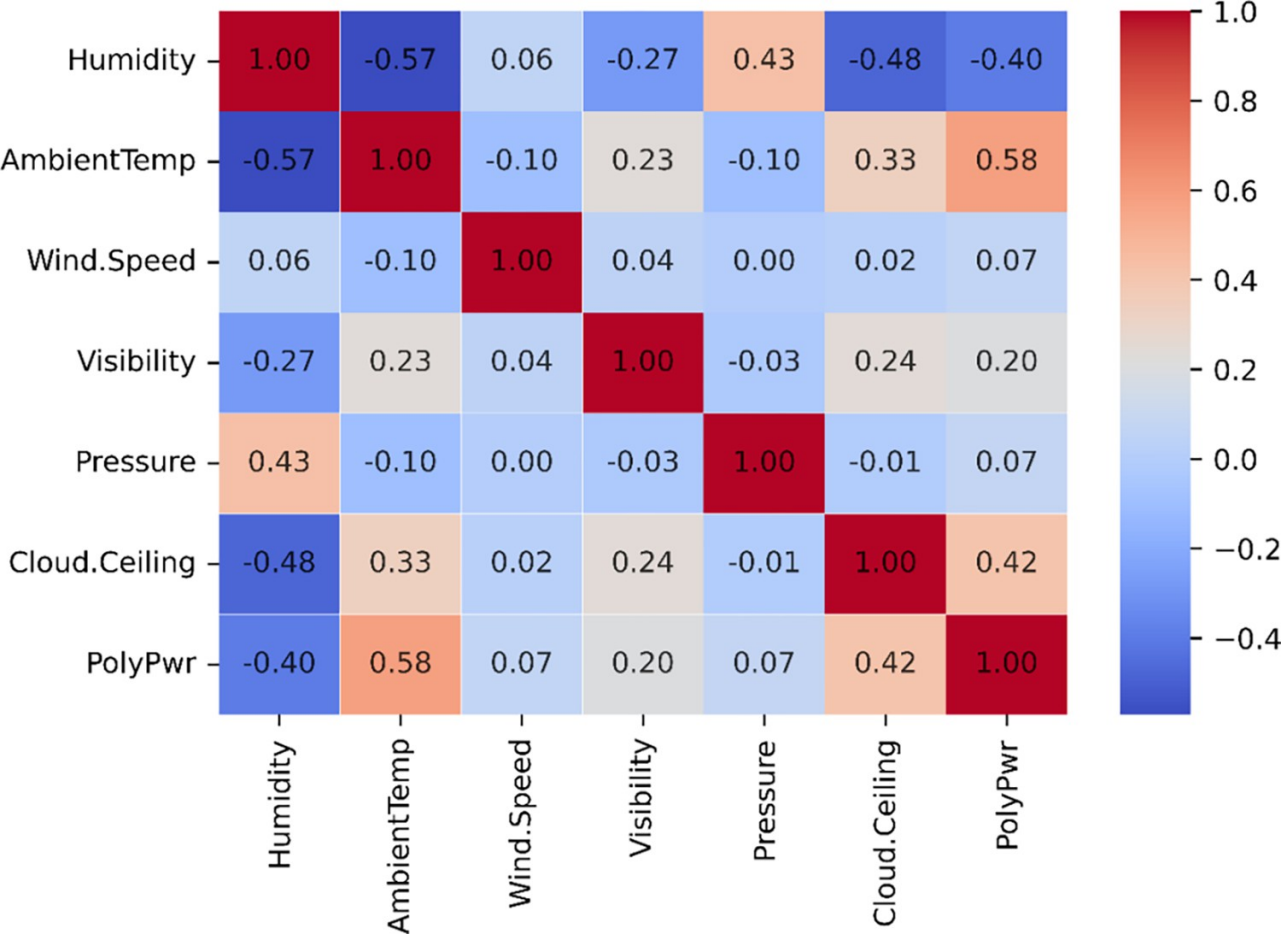
Correlation matrix analysis input variables in this study.

Fig 3 depicts the linear correlation among the variables of the dataset used. It can be observed that the input variables exhibit very little correlation. Only variable Ambient temperature has the highest correlation value, which is 0.58.

In order to complete the description of correlation analysis, Table 2 summarizes the variance inflation factor (VIF) values for six variables, providing insights into multicollinearity within the dataset. All variables, including Humidity (2.360), Ambient Temperature (1.570), Wind Speed (1.020), Visibility (1.117), Cloud Ceiling (1.401), and Pressure (1.364), exhibit VIF values well below the threshold of concern (commonly 5 or 10). This indicates minimal multicollinearity, ensuring that no variable excessively influences others. Consequently, the dataset is suitable for regression analysis without requiring corrective measures for multicollinearity.

**Table 2:** Multicollinearity using the variance inflation factor (VIF).

Variable	Humidity	Ambient Temperature	Wind speed	Visibility	Cloud ceiling	Pressure
VIF	2.360	1.570	1.020	1.117	1.401	1.364

## 4. Machine learning approach

### 4.1. Gradient Boosting Regressor (GB)

Gradient Boosting is a powerful machine learning algorithm that enhances predictive performance by combining the outputs of multiple weak learners, typically decision trees, to create a single strong model. It is an iterative algorithm where each subsequent model is trained to correct the errors made by the previous models [39]. Gradient Boosting is particularly effective in scenarios with complex data patterns and when high predictive accuracy is required. It can handle both regression and classification tasks and is known for its ability to reduce bias and variance, leading to robust models.

The process begins by fitting the first model to the data, which is usually a simple decision tree. The predictions from this model are then compared to the actual values, and the difference, or residuals, is calculated. The next model is trained on these residuals, with the aim of reducing the error made by the first model. This process is repeated for a specified number of iterations, with each model learning to improve upon the errors of the combined model from the previous iteration. The core idea behind Gradient Boosting is to minimize a loss function, which measures the difference between the actual and predicted values, by sequentially fitting models to the residual errors of the combined model. One of the key aspects of Gradient Boosting is its use of gradient descent, a numerical optimization technique, to minimize the loss function. In each iteration, the algorithm calculates the gradient of the loss function with respect to the predictions and updates the model in the direction that reduces the loss. This approach allows the model to progressively "boost" its performance by focusing on the areas where it is weakest. However, it is computationally intensive and can be prone to overfitting if not properly tuned, requiring careful management of parameters like learning rate, the number of trees, and tree depth.

### 4.2. Extreme Gradient Boosting Regressor (XGBoost)

The XGBoost model operates by constructing a sequence of decision trees, where each tree learns from the errors of its predecessors [40]. During this process, XGBoost computes gradients of the objective function (typically the loss function) and utilizes these gradients to update the decision values. This iterative process continues until a specified number of iterations or stopping conditions are met.

XGBoost is a powerful and widely used machine learning algorithm within the machine learning community. It inherits and extends upon previous algorithms such as Gradient Boosting Machine (GBM) and is particularly suited for predicting complex structured data, especially in areas like ensemble learning, time series forecasting, and natural language processing [41]. Some advantages of the XGBoost model include: high performance, scalability to large datasets, flexibility, and fine-tuning of parameters.

### 4.3. K-neighbors Regressor (KNN)

The k-Nearest Neighbor (KNN) algorithm is a widely used supervised learning method, notable for its simplicity and effectiveness [42]. It is often listed among the top data mining algorithms due to its intuitive approach to classification and regression tasks. KNN creates a decision boundary that closely follows the distribution of the data, which helps in achieving high accuracy when the dataset is large and representative.

KNN is a nonparametric algorithm, meaning it does not assume any specific form for the underlying data distribution. This characteristic makes it particularly suitable for real-world datasets that may not adhere to theoretical distributions like Gaussian mixtures or linear separability [43]. Nonparametric methods like KNN can handle a variety of data distributions more effectively. Unlike other algorithms that build a model during the training phase, KNN has a minimal training phase and a more intensive testing phase. During training, KNN simply stores the dataset, while during testing, it classifies new data points by examining the ’k’ nearest neighbors from the stored dataset. This means that while the training process is fast, the algorithm requires access to the entire training dataset (or a significant portion of it) during the prediction phase, making the testing phase more computationally demanding [44].

### 4.4. Light Gradient Boosting Machine Regressor (LightGBM)

The Light Gradient Boosting Machine (LightGBM) is an advanced machine learning algorithm that has gained popularity for its efficiency and high performance in both classification and regression tasks [45]. Developed by Microsoft, LightGBM is designed to be highly efficient and scalable, capable of handling large datasets with substantial features while maintaining rapid training and prediction times.

Another distinctive feature of LightGBM is its leaf-wise (or best-first) tree growth strategy, as opposed to the level-wise approach used by many other gradient boosting algorithms. In the leaf-wise method, LightGBM grows trees by splitting the leaf with the highest loss, which can result in deeper trees with fewer splits, leading to better accuracy and efficiency. Overall, LightGBM stands out due to its speed, scalability, and accuracy, making it a preferred choice for many machine learning practitioners dealing with large-scale datasets and complex predictive tasks [46].

### 4.5. CatBoost Regressor (CatBoost)

CatBoost, short for Categorical Boosting, is a high-performance machine learning algorithm developed by Yandex [47]. It excels in both classification and regression tasks. CatBoost is designed to handle categorical data without extensive preprocessing, making it a powerful tool for real-world applications where such data is prevalent.

One of the standout features of CatBoost is its ability to directly incorporate categorical features into the model. While traditional gradient boosting algorithms often require categorical data to be converted into numerical format (e.g., one-hot encoding), CatBoost can directly process categorical variables, maintaining their inherent information and relationships. This is achieved through a process called target-based encoding, where the algorithm replaces categorical values with statistics computed from the target variable. CatBoost also implements efficient processing techniques to speed up training and inference. These include sophisticated algorithms for efficient memory and computational resource usage, making CatBoost suitable for large-scale datasets [48].

### 4.6. Performance indicators

During the evaluation process of machine learning models, employing evaluation methods such as R^2^, mean absolute error (MAE) and root mean square error (RMSE) offer detailed insights into the model’s predictive performance across both training and validation datasets.

The R^2^ coefficient of determination, quantifies how much of the variation in the dependent variable can be explained by the independent variables. It has a range from 0 to 1, with a value of 1 representing a perfect fit.

R2=1−∑i=1n(yi−yi^)2∑i=1n(yi−yi−)2 (1)

Where, y_i_ represents the actual value, yi^ represents the predicted value by the model for the i sample, n is the number of samples in the test dataset, and $y_{i}^{-}$ is the mean of the actual values y_i_

The mean absolute error (MAE) represents the average of the absolute differences between predicted values and actual values. It provides a straightforward measure of prediction accuracy by calculating the average magnitude of errors in a set of predictions, without considering their direction. Lower MAE values indicate better predictive accuracy, as they signify smaller errors between the predicted and actual values.


MAE=1n∑i=1n⌊yi−yi^| (2)

The root mean square error (RMSE) measures the average magnitude of the errors between predicted and actual values in a dataset. A lower RMSE indicates better predictive accuracy, while a higher RMSE suggests larger prediction errors.


RMSE=1n∑i=1n(yi−yi^)2 (3)

Additionally, using cross-validation techniques is an important method to evaluate model generality. By dividing the data into subsets, the model is trained on one subset and evaluated on the remaining subset. The results provide insight into the model’s average performance across multiple test datasets, supporting the assessment of the model’s overall robustness and stability.

### 4.7. Methodology flow chart

**Fig 4:**
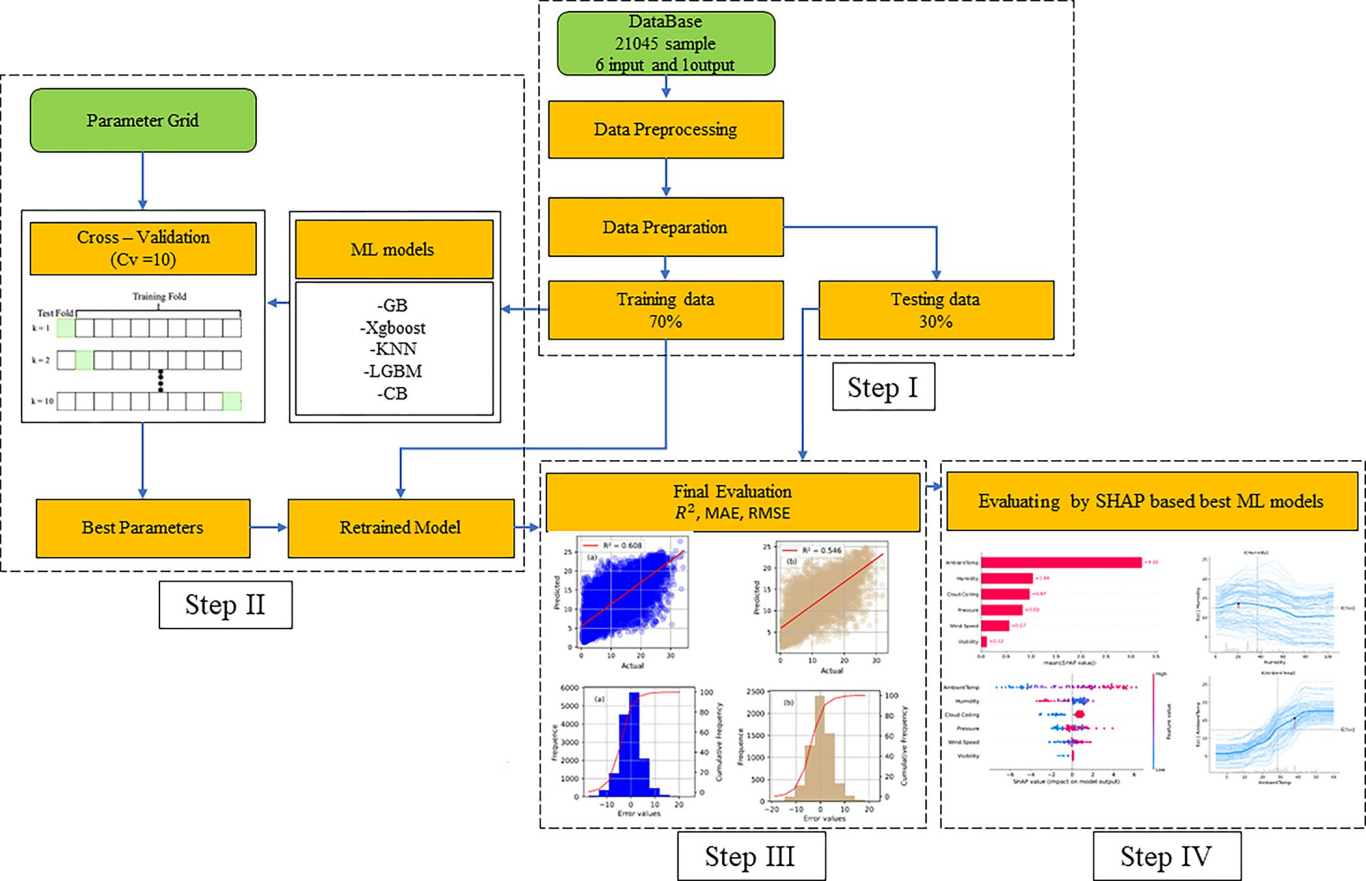
Methodology flow chart of machine learning model in this investigation.

Fig 4 “Methodology flow chart” describes the main steps including 4 steps in this investigation using ML models for forecasting solar energy generated by photovoltaic panel. The ML models of this investigation are implemented by Python language programing with Sklearn library [49].

- Step I: Data Preparation

The dataset utilized in this study comprises a total of 21,045 samples, each containing 6 input features including Humidity, temperature, wind speed, visibility, cloud ceiling, pressure, and 1 output feature “solar energy”. The input features represent various environmental, while the output feature corresponds to the power generation capacity of solar cells. To ensure that the data is in a suitable form for model training, comprehensive data preprocessing is carried out across the entire dataset. This preprocessing includes tasks such as data cleaning and normalization or standardization of features by Sklearn library [49].

Once the dataset is fully preprocessed, it is then divided into two subsets: training data and testing data. The training data, which constitutes 70% of the entire dataset, is used for building and fine-tuning the machine learning models. The remaining 30% of the dataset is reserved for testing and validating the performance of the models. This split ensures that the models are trained on a large portion of the data while still leaving a significant amount of data for unbiased evaluation.

- Step II: Model Training

In the model training phase, a systematic approach is taken to optimize the performance of the selected machine learning models. A parameter grid is established for each model, specifying a range of hyperparameters that will be tuned to find the optimal configuration. This process is critical as the choice of hyperparameters can significantly impact the performance of the models.

To ensure that the models generalize well to unseen data, cross-validation is employed during training. Specifically, 10-fold cross-validation (CV = 10) is used, meaning that the training data is divided into 10 subsets. Each model is trained 10 times, with each iteration using a different subset as validation data while the remaining subsets are used for training. This method helps to mitigate overfitting and provides a more reliable estimate of model performance.

The machine learning models under consideration include Gradient Boosting (GB), XGBoost, K-Nearest Neighbors (KNN), LightGBM (LGBM), and CatBoost (CB). After the cross-validation process is complete, the model with the best performance for each algorithm is identified based on the cross-validation results. The best parameters identified during this process are then used to retrain the model on the entire training dataset, ensuring that the model is optimized before final evaluation.

- Step III: Model Evaluation

Once the models have been trained and retrained with the best parameters, they are subjected to a thorough evaluation process to assess their predictive performance. This evaluation is carried out using a set of commonly used metrics that provide insights into different aspects of model performance. The coefficient of determination (R^2^) is used to measure how well the model’s predictions match the actual values. A higher R^2^ indicates better predictive accuracy.

Additionally, Mean Absolute Error (MAE) and Root Mean Squared Error (RMSE) are calculated to evaluate the models’ accuracy in predicting the output. MAE provides a measure of the average magnitude of errors in predictions, while RMSE gives more weight to larger errors, making it a useful metric when large deviations from actual values are of particular concern.

To complement these numerical evaluations, various visualizations are generated to provide a more intuitive understanding of the models’ performance. Scatter plots will be used to compare predicted values against actual values, highlighting the accuracy and potential biases in the predictions. Histograms can be utilized to visualize the distribution of errors, allowing for a deeper understanding of how the models perform across different ranges of the data.

- Step IV: SHAP-based Evaluation

After the initial evaluation, the best-performing machine learning models undergo a further, more detailed analysis using SHAP (SHapley Additive exPlanations). SHAP is a powerful method for interpreting complex models by breaking down the prediction of each sample into contributions from each feature. This analysis helps to understand how each input feature influences the model’s predictions, providing insights into the relationships between input features and the output.

The SHAP values are visualized through various plots, such as SHAP summary plots and dependence plots, which help in interpreting the model’s behavior. By understanding the contribution of each feature, it becomes possible to explain the model’s decisions, identify key drivers of the output, and gain confidence in the model’s predictions. This interpretability is crucial, especially in applications like solar energy forecasting, where understanding the factors influencing predictions can lead to better decision-making and model trustworthiness.

## 5. Evaluation of machine learning model

The dataset is divided into two sets: a training set and a validation set, with a split ratio of 70%/30%. The validation set aids the algorithm in building a machine learning model based on the values of hyperparameters available in the Sklearn library.

Table 3 summarizes the optimal hyperparameters for five machine learning models tuned using GridSearchCV and Bayesian Optimization. For Gradient Boosting (GB) and LightGBM, both approaches converged to similar n_estimators (500) but differed in learning rates, with Bayesian Optimization favoring finer adjustments (learning_rate: 0.001). CatBoost demonstrated a notable difference in depth (depth: 10 for GridSearchCV vs. 4 for Bayesian Optimization). KNN showed slight variation in n_neighbors, favoring 21 under Bayesian Optimization.

**Table 3:** Optimal hyperparameters for each machine learning model after using GridsearchCV and Bayesian Optimization.

No	Model	Best_params Gridsearchcv	Best_params Bayesian Optimization
1	GB	{’n_estimators’: 500, ’learning_rate’: 0.01}	{’n_estimators’: 500, ’learning_rate’: 0.01}
2	XGBoost	{’learning_rate’: 0.01, ’n_estimators’: 500}	{’learning_rate’: 0.001, ’n_estimators’: 500}
3	KNN	{’n_neighbors’: 20, ’weights’: ’uniform’}	{’n_neighbors’: 21, ’weights’: ’uniform’}
4	LGBM	{’learning_rate’: 0.01, ’n_estimators’: 500}	{’learning_rate’: 0.001, ’n_estimators’: 500}
5	CatBoost	{’depth’: 10, ’learning_rate’: 0.01, ’iterations’: 500}	{’depth’: 4, ’learning_rate’: 0.01, ’iterations’: 500}

Table 4 evaluates the models’ predictive performance. CatBoost consistently achieved the best R^2^, lowest MAE, and RMSE across training and testing, highlighting its robustness. Gradient Boosting and LightGBM showed improved R^2^ and RMSE with Bayesian Optimization, confirming the benefit of fine-tuning. However, KNN maintained a stable performance between both methods. Notably, XGBoost showed no significant improvement in R^2^, indicating potential limitations in hyperparameter exploration. Upon reviewing the performance metrics, GridSearchCV results indicate that CatBoost achieved the highest testing R^2^ = 0.546, outperforming Bayesian Optimization’s R^2^ of 0.538. This suggests that the hyperparameters derived from GridSearchCV were better tuned for the model under this dataset. For testing MAE and RMSE, CatBoost still displayed strong results under GridSearchCV, with MAE of 3.583 W and RMSE of 4.748 W, both comparable to Bayesian Optimization results. This emphasizes CatBoost’s capability to maintain high predictive accuracy across various evaluation metrics.

**Table 4:** The summary table of performance metrics for the five algorithms.

No	Parameters	GridsearchCV		Bayesian Optimization	
		Training	Testing	Training	Testing
R^2^					
1	Gradient Boosting	0.400	0.392	0.553	0.526
2	XGBoost	0.468	0.442	0.468	0.442
3	KNN	0.572	0.525	0.572	0.531
4	LightGBM	0.453	0.436	0.453	0.436
5	CatBoost	0.608	0.546	0.623	0.538
MAE (W)					
1	Gradient Boosting	4.640	4.604	3.641	3.703
2	XGBoost	4.355	4.381	4.355	4.381
3	KNN	3.490	3.642	3.500	3.607
4	LightGBM	4.407	4.399	4.407	4.398
5	CatBoost	3.367	3.583	3.293	3.596
RMSE (W)					
1	Gradient Boosting	5.542	5.497	4.785	4.489
2	XGBoost	5.219	5.263	5.219	5.264
3	KNN	4.679	4.859	4.681	4.825
4	LightGBM	5.290	5.292	5.290	5.291
5	CatBoost	4.478	4.748	4.396	4.786

**Fig 5:**
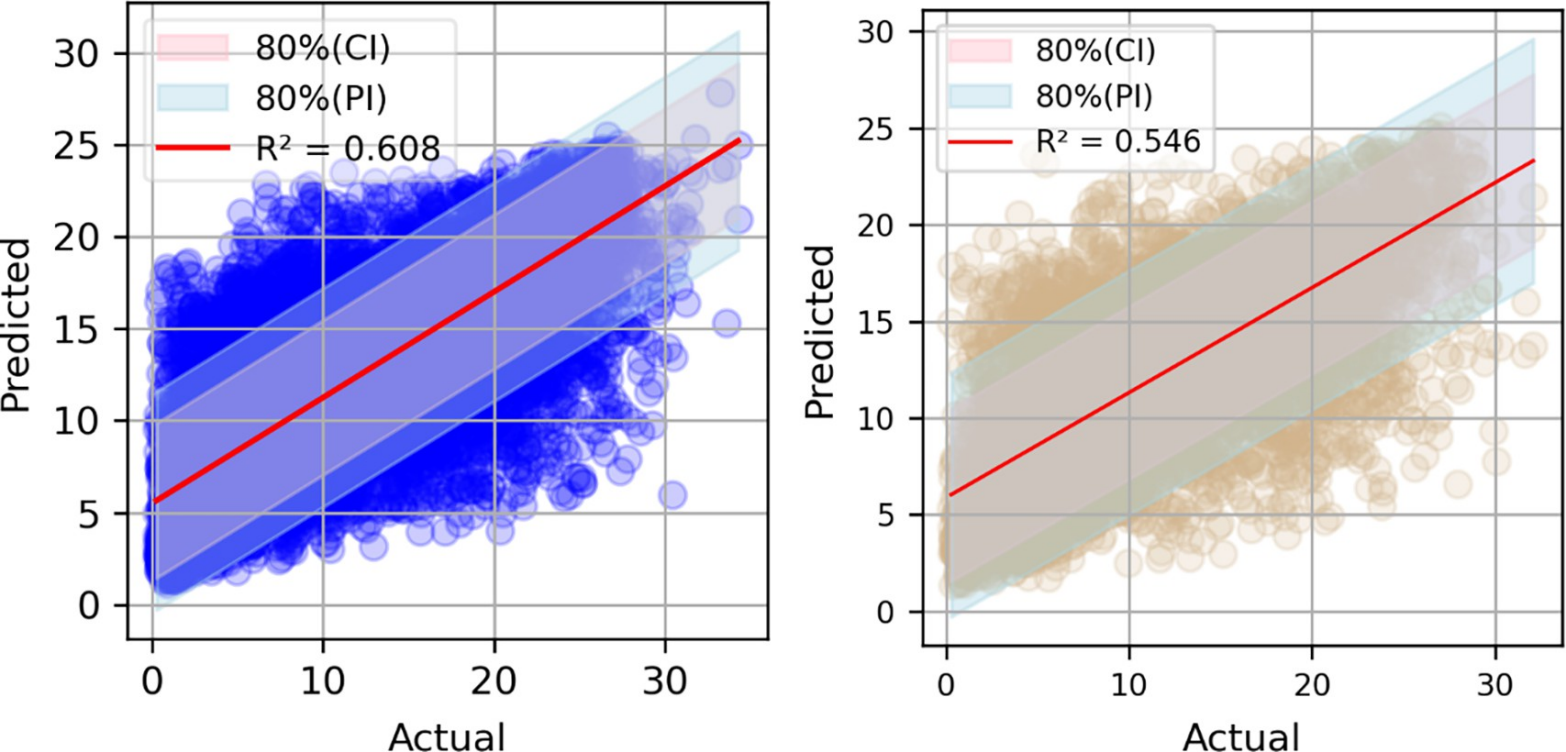
Correlation between true value and Catboost predicted value of solar energy with (a) training dataset, (b) testing dataset.

Fig 5 illustrates the correlation between the true values and the predicted values of solar energy using the CatBoost model for (a) the training dataset and (b) the testing dataset. The red line represents the best-fit line, while the shaded regions depict the 80% Confidence Interval (CI) and the 80% Prediction Interval (PI). For the training dataset, the number of points within the 80% CI is 12,199 and the number of points within the 80% PI is 13,743. For the testing dataset, the number of points within the 80% CI is 5,166 and the number of points within the 80% PI is 5,882.

The left plot (training dataset) shows a higher R^2^ value 0.608 compared to the right plot (testing dataset), which has an R^2^ value of 0.546. This indicates that the model performs slightly better on the training dataset than on the testing dataset. Both plots highlight the model’s ability to predict solar energy with varying confidence and prediction intervals.

The predictive performance of the CatBoost model is not really robust, that implies suggesting a significant influence of the quality and quantity data on the predicted value. That will be more discussed in the following section.

**Fig 6:**
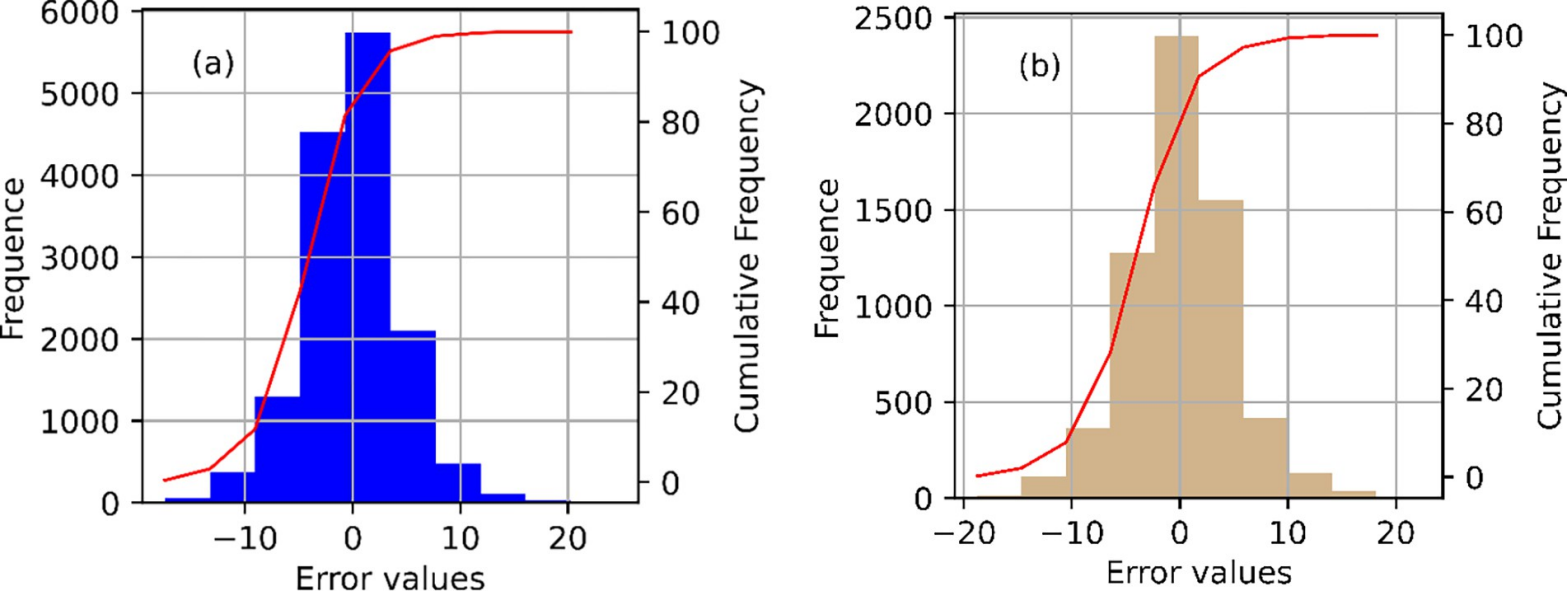
RMSE values with (a) training, (b) testing.

Fig 6A and 6B illustrates the comparison between predicted and actual solar panel energy values, primarily distributed within the ±10% error range, with a significant number of points lying along the y = x line. Therefore, the prediction error values between the model-predicted solar panel energy and the actual solar panel energy of the training dataset, as depicted in Fig 6, are mainly distributed within the range of ±10 W.

To gain a deeper understanding of the CatBoost model’s ability to forecast solar energy, the SHAP model will be employed to interpret the influence of input variables on the prediction of solar energy. The findings of this analysis will be presented in the following section.

## 6. SHAP-based evaluation

The SHAP (Shapley Additive exPlanation) framework, rooted in cooperative game theory as introduced by Lundberg and Lee [50], was initially designed to quantify individual contributions in cooperative games. Since then, SHAP has evolved into a powerful tool for interpreting machine learning model predictions [51]. By integrating various existing interpretability methods, SHAP offers an intuitive, theoretically sound approach for explaining model outputs, representing a major advance in the field of model interpretation. Central to this framework are SHAP values, which provide detailed insights into the magnitude and direction (positive or negative) of feature influences on model predictions. These values are essential for understanding the relative importance of different features in shaping model outcomes.

One of the key visualization tools in SHAP is the summary plot, which combines both feature importance and their effects on predictions. Each point on the plot corresponds to a SHAP value for a particular feature and instance, with the y-axis denoting the feature and the x-axis representing the SHAP value. The color gradient of the points reflects the feature values, from low to high. To address overlapping data points, jittering is applied along the y-axis, visually representing the distribution of Shapley values for each feature. Features are typically ordered by importance, making it easier to identify key drivers of model predictions. While the summary plot provides an initial understanding of the relationship between feature values and their predictive impact, more detailed insights can be gained from SHAP dependence plots, which will be discussed further.

### 6.1. SHAP global interpretation

**Fig 7:**
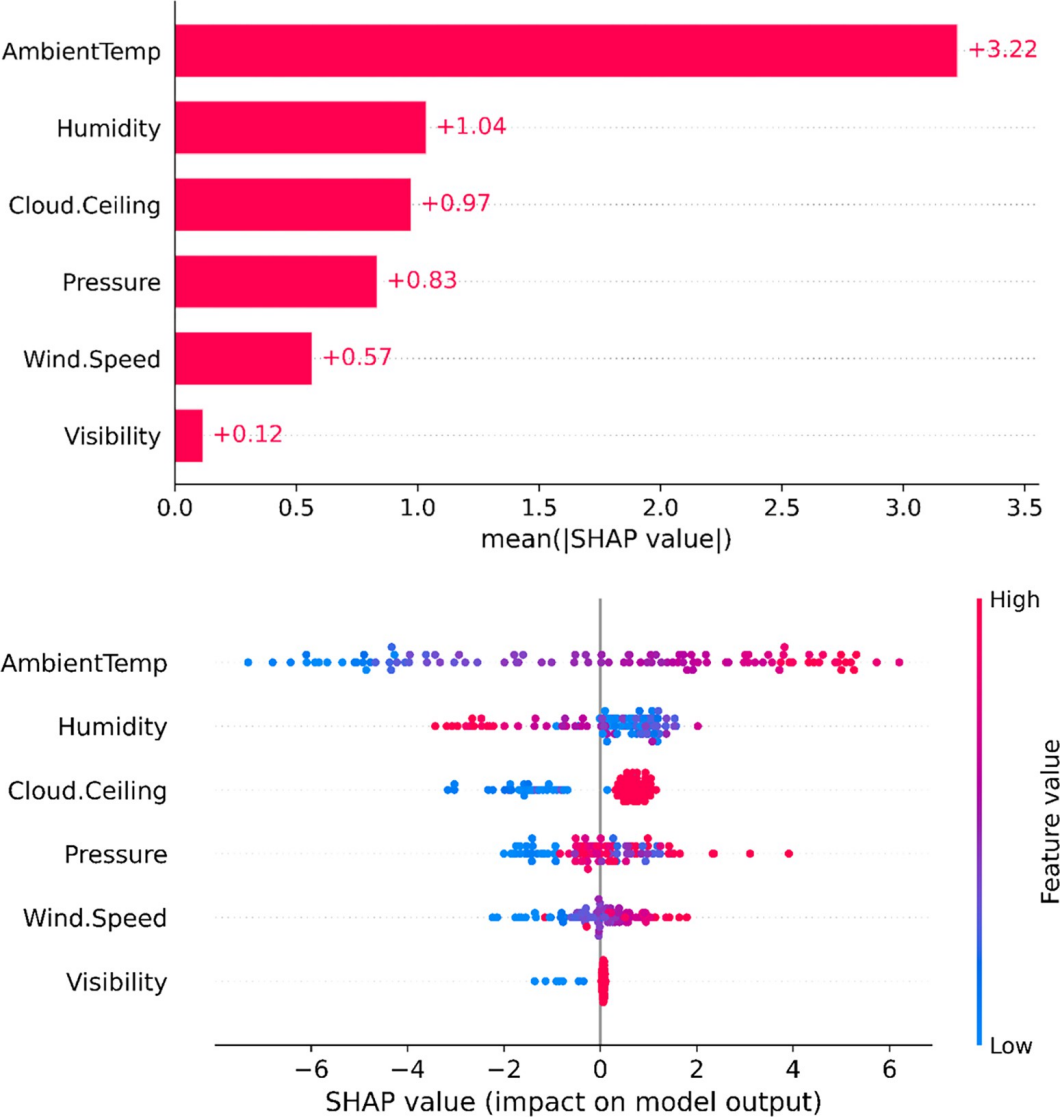
SHAP global interpretation feature importance of each input variable on predicted value of solar energy (a) absolute mean SHAP value, and (b) global SHAP value.

Fig 7 presents the SHAP global interpretation of feature importance for each input variable’s effect on the predicted value of solar energy, with (a) showing the absolute mean SHAP values and (b) displaying the global SHAP values. The results in Fig 7A indicate that Ambient temperature has the most significant impact on the accuracy of solar energy predictions made by CatBoost, followed by Humidity, Cloud ceiling, Pressure, Wind speed, and Visibility. This ranking reflects the relative importance of each feature in explaining the variability in the model’s predictions.

Meanwhile, the SHAP values shown in Fig 7B illustrate the specific influence of each input variable on the predicted solar energy value. Specifically, higher Ambient temperatures lead to an increase in solar energy production, with the impact ranging approximately ±6W. Lower Humidity levels are favorable for generating solar energy from photovoltaic panels, with the influence ranging from -4W to +2W. Similarly, higher values of Cloud ceiling, Pressure, Wind speed, and Visibility generally enhance solar energy production. However, Visibility has a relatively minor effect on the variation of the global SHAP value compared to the other factors.

To provide a more detailed quantitative assessment of the influence of each input variable on the predicted solar energy value, the Partial SHAP dependence values for each input variable will be described in the following section. In particular, SHAP local value analysis will help analyze errors, as well as improve the accuracy of solar energy prediction models.

### 6.2. Partial SHAP dependence interpretation

**Fig 8:**
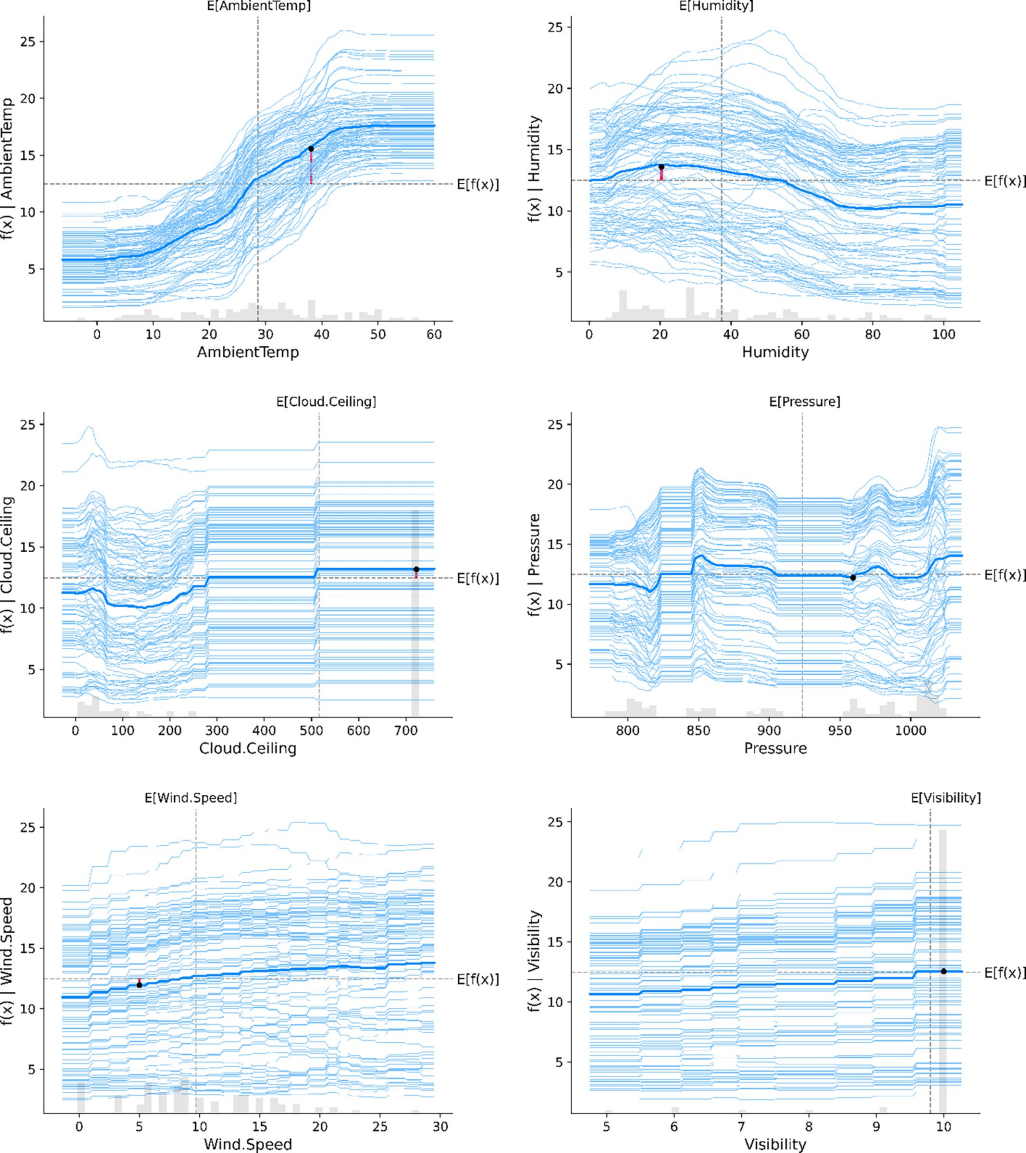
Partial SHAP dependence plot of the six representative features.

By examining the Partial SHAP dependence plots for these features shown in Fig 8, we can gain a deeper understanding of how changes in their values affect the model’s predictions. This analysis helps us uncover the exact form of the relationship between each feature and the model output, including any non-linearities or interactions with other features.

The results of the Partial SHAP dependence shown in Fig 8 reveal some interesting insights into the impact of the six input variables on solar energy production. The statistical analysis indicates that the average value for the 21045 solar energy samples is around 13 W, which corresponds to the intersection of the E(input variable) and f(x)|(input variable) lines. The Partial dependence plot aligns with the results depicted in the Global SHAP value in Fig 7B. Specifically, the Partial SHAP dependence curves for Cloud Ceiling (cf. Fig 8C), Pressure (cf. Fig 8D) and Visibility (cf. Fig 8F) show relatively small changes, hovering close to the average solar energy value, indicating that these three variables have minimal influence on solar energy production.

In contrast, the impact curves for Wind Speed (cf. Fig 8E), Humidity (cf. Fig 8B), and Ambient Temperature (cf. Fig 8A) on solar energy are non-linear, particularly for Ambient Temperature. Solar energy values increase almost linearly with Wind Speed, demonstrating a more significant and direct influence on energy production.

### 6.3. SHAP local interpretation

**Fig 9:**
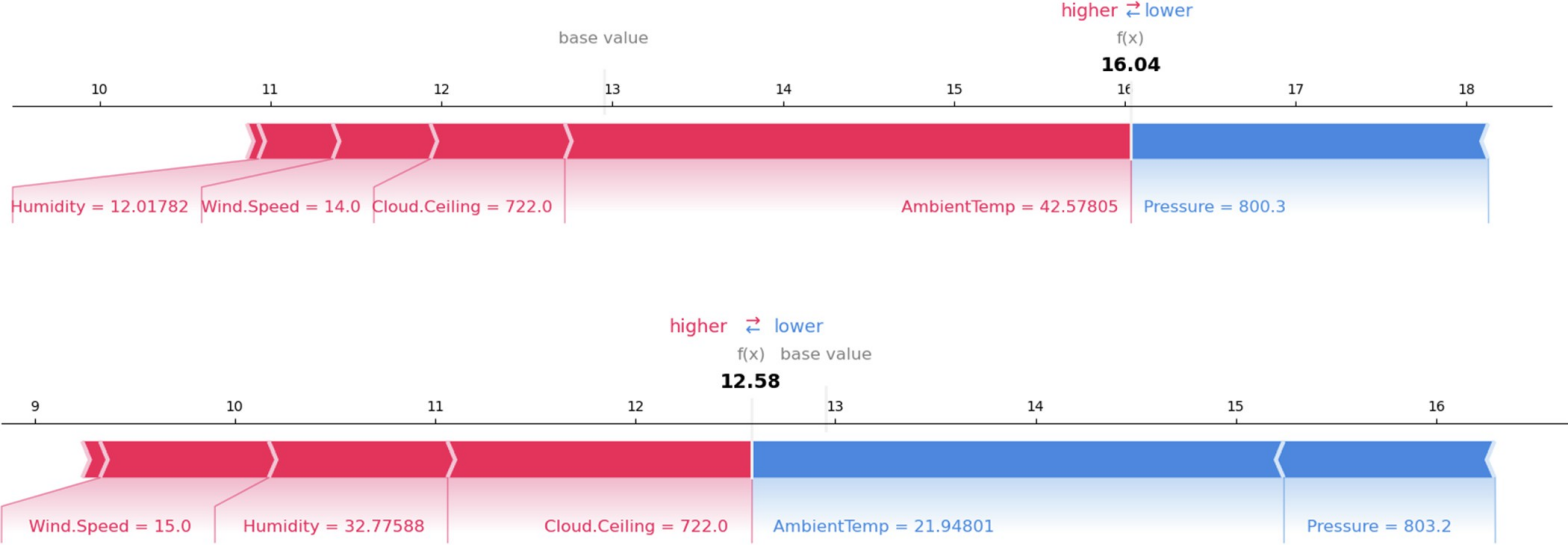
SHAP local value of two specific cases. (a) Actual value 18.33 W vs Predicted value 16.04 W of solar energy. (b) Actual value 12.42 W vs Predicted value 12.58 W of solar energy.

Fig 9 illustrates the specific impact of each input variable on the predicted solar energy values using SHAP local value analysis. This analysis is applied to two actual solar energy values: 18.33 W and 12.42 W, with corresponding predicted values of 16.04 W and 12.58 W (shown in Fig 9A and 9B). The prediction errors depicted in Fig 6 can be partly explained by the results presented in Fig 9.

In this study, six input variables contribute to the solar energy predictions. Among these, Ambient temperature and Humidity have the most significant influence on the model’s accuracy and prediction capabilities. Consequently, the prediction errors are primarily attributed to the variability in these two input variables.

Additionally, solar energy output depends on the technical specifications of the photovoltaic panels, which were not included in the database used for model development (S1 Data). The absence of these panel-specific variables across the 21,045 samples from different locations contributes to the prediction errors observed in this study.

However, using the six weather-related input variables still allows for a simplified preliminary feasibility prediction for solar energy projects. This approach is especially useful during the initial assessment phase when specific technical details of the photovoltaic panels are not yet available.

**Fig 10:**
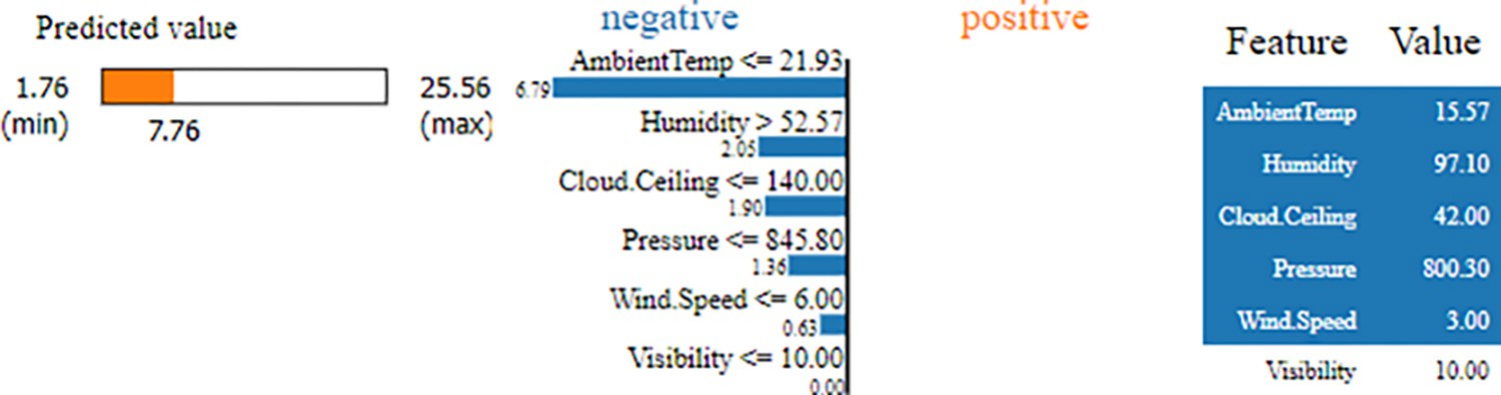
LIME providing local interpretation of the specific case with true value 30.058 W comparing with predicted value 7.764 W.

Fig 10 demonstrates the use of LIME (Local Interpretable Model-agnostic Explanations) to analyze a specific case where the true value is 30.058 W, while the model predicts 7.764 W, indicating significant underprediction. The predicted value lies within a range of 1.76 W to 25.56 W, with the orange bar highlighting the prediction. The analysis identifies key features influencing the prediction, with Ambient Temperature (≤ 21.93) having the largest negative impact (6.79), followed by Humidity (> 52.57) (2.05) and other factors such as Cloud Ceiling (≤ 140.00), Pressure (≤ 845.80), Wind Speed (≤ 6.00), and Visibility (≤ 10.00). The actual feature values—Ambient Temperature: 15.57, Humidity: 97.10, Cloud Ceiling: 42.00, Pressure: 800.30, Wind Speed: 3.00, and Visibility: 10.00—help explain the model’s low prediction.

LIME reveals the specific reasons behind the poor performance of the model for this case. Key features such as low Ambient Temperature (15.57) and high Humidity (97.10) strongly influence the underprediction. These insights can guide further investigation into feature interactions or the model’s handling of extreme cases, ultimately improving its performance.

## 7. Discussion and limitation

The regression results achieved in this study reveal certain limitations in the predictive capability of the machine learning models, as indicated by the relatively modest R^2^ values throughout the experiment. These findings suggest latent complexities in the relationship between the input variables (such as ambient temperature, humidity, and wind speed) and the target variable (solar energy output), which the models were unable to fully capture.

A primary reason for the lower R^2^ values is the inherent variability and non-linear interactions between solar energy output and its influencing factors. While the input features provide a preliminary basis for prediction, they do not comprehensively account for all the variables that govern solar energy production. Notably, the absence of photovoltaic panel-specific technical specifications such as panel efficiency, orientation, and degradation rates likely introduced significant noise into the predictions. These variables are critical for accurately modeling solar energy output but were unavailable in the dataset.

Another contributing factor is the dataset’s inherent characteristics. The data used in this study comprises 21045 samples derived from a limited geographical and technological context, which may have introduced biases. For instance, if the dataset predominantly represents regions with specific weather patterns or solar radiation profiles, the trained models may struggle to generalize to broader or more diverse conditions. Furthermore, discrepancies in data quality or measurement precision could also have influenced the results, particularly for features like cloud ceiling and visibility, which exhibit lower importance in the SHAP analysis.

Despite these challenges, the study offers valuable insights into the potential of machine learning models for solar energy forecasting. While the achieved R^2^ values suggest limited accuracy for precise predictions, the models remain useful for initial feasibility assessments of solar farm locations. These assessments rely on readily available weather data and provide a foundation for further analysis.

To enhance model performance and reliability, future research should address the following:

1. Data Enrichment: Collecting larger, more diverse datasets that incorporate detailed technical specifications of photovoltaic panels, as well as broader environmental conditions across different geographic regions.
2. Feature Exploration: Identifying additional relevant features, such as solar panel tilt angles, shading effects, or maintenance schedules, to capture more nuanced relationships between inputs and outputs.
3. Model Refinement: Leveraging advanced machine learning techniques, such as ensemble methods or hybrid models, to improve the capture of non-linearities and complex feature interactions.

Ultimately, addressing these limitations will enhance the accuracy and applicability of predictive models, enabling more effective integration of solar energy into the grid and supporting the global transition to sustainable energy sources.

## 8. Conclusion

This study highlights the potential and challenges of using five machine learning models, particularly the highest performance of CatBoost model with training values of R^2^ value of 0.608, RMSE of 4.478 W and MAE of 3.367 W and the validation value is R^2^ of 0.46, RMSE of 4.748 W and MAE of 3.583 W, for solar energy prediction. The integration of weather-related input variables, such as temperature, humidity, wind speed, and visibility, provides a foundation for preliminary feasibility assessments of solar farm locations. However, the limited performance of the models, as demonstrated by the R^2^, RMSE and MAE values, suggests that the relationship between the input variables and solar energy output is more complex than captured by the current dataset and models. The lack of photovoltaic panel-specific technical data likely contributed to prediction errors, emphasizing the need for more comprehensive datasets that include both weather conditions and system specifications.

The SHAP analysis offered valuable insights into the contribution of each feature to the predictions, with ambient temperature and humidity emerging as the most influential factors. The Partial SHAP dependence plots revealed non-linear interactions, particularly for variables like wind speed and temperature, further demonstrating the intricacies involved in accurately predicting solar energy output.

While the study underscores the importance of leveraging modern machine learning techniques, it also highlights key limitations, including dataset biases related to geographical focus and technological constraints. Future research should aim to collect larger, more diverse datasets, incorporating a wider range of environmental conditions and solar technologies to enhance model performance and generalizability. Ultimately, these advancements will improve the accuracy of solar energy forecasting, supporting the effective integration of renewable energy into the power grid.

## Supporting information

S1 Data (CSV)
